
JOURNAL INFORMATION
==============================
NLM Title Abbreviation: J Diabetes Investig
Journal ID: J Diabetes Investig
NLM Journal ID: 101520702
Journal ID: JDI

Journal of Diabetes Investigation

ISSN: 2040-1116
EISSN: 2040-1124
Publisher: Wiley

ARTICLE INFORMATION
==============================
PMCID: PMC4906256
DOI: 10.1111/jdi.12507
Article ID: JDI12507
Article version: 1
Subjects: Scientific Programme; Abstracts from The 7th Scientific Meeting of the Asian Association for the Study of Diabetes and the Annual Scientific Meeting of the Hong Kong Society of Endocrinology, Metabolism and Reproduction, 21‐22 November 2015, Hong Kong, China; Scientific Programme

Scientific Programme

Electronic publication date: 2016 Jun 14
Print publication date: 2016 May
Volume: 7
Issue: Suppl 2
Issue title: Abstracts from The 7th Scientific Meeting of the Asian Association for the Study of Diabetes and the Annual Scientific Meeting of the Hong Kong Society of Endocrinology, Metabolism and Reproduction, 21‐22 November 2015, Hong Kong, China
First page: 5
Last page: 8
Copyright: © 2016 The Authors. Journal of Diabetes Investigation published by Asian Association for the Study of Diabetes (AASD) and John Wiley & Sons Australia, Ltd
License: This is an open access article under the terms of the Creative Commons Attribution License, which permits use, distribution and reproduction in any medium, provided the original work is properly cited.
License URL: https://creativecommons.org/licenses/by/4.0/

Page count: 4
source-schema-version-number: 2.0
component-id: jdi12507
cover-date: May 2016
details-of-publishers-convertor: Converter:WILEY_ML3GV2_TO_NLMPMC version:4.9.1 mode:remove_FC converted:14.06.2016

==============================
### SCIENTIFIC PROGRAMME

### 21 November (Saturday)

**Venue: N101-112, Level 1, Hong Kong Convention and Exhibition Centre (HKCEC)**

**08:30 – 09:30**

Registration — *N101 Foyer*

**09:30 – 10:30**

**Oral Presentations (Basic)** — *N101B*
*Chairpersons:*
Prof. Hyeong-Kyu PARK (Korea)
Prof. Aimin XU (Hong Kong)

**Oral Presentations (Clinical)** — *N101A*
*Chairpersons:*
Dr. Andrea LUK (Hong Kong)
Prof. Naoko TAJIMA (Japan)

**10:30 – 11:00**

Health Care Industry Exhibition and Coffee Break — *N106-108*

Poster Viewing — *N109-112*

**11:00 – 11:30**

**Opening Ceremony** — *N101B*
*Guest of Honour:* Dr. Wing-Man KO, BBS, JP
*Secretary for Food and Health, The Government of the Hong Kong Special Administrative Region*

**11:30 – 12:30**

**Plenary Lecture 1: The Yutaka Seino Distinguished Leadership Award Presentation and Lecture** — *N101B*
*Chairpersons:* Prof. Ronald MA (Hong Kong), Prof. Susumu SEINO (Japan)

**Canary in the Coal Mine**
Prof. Wilfred FUJIMOTO
*School of Medicine, University of Washington, USA*

**12:30 – 13:00**

Lunch — *N101B*

Poster Viewing — *N109-112*

**13:00 – 13:50**

**Lunch Symposium (Sponsored by Boehringer Ingelheim)** — *N101B*
*Chairperson:* Dr. Peter TONG (Hong Kong)

**SGLT2-inhibitors: A Novel Class for the Treatment of Type 2 Diabetes**
Prof. Kathryn TAN
*Department of Medicine, The University of Hong Kong, Hong Kong*

**EMPA-REG OUTCOME™ – Implications for Management of Patients with Type 2 Diabetes**
Prof. Per-Henrik GROOP
*University of Helsinki, Finland*

**14:00 – 15:15**

**Symposium 1** — *N101B*
**The Beta-Cell and Insulin Secretion**
*Chairpersons:*
Prof. Hong-Kyu LEE (Korea)
Dr. Jenny LEUNG (Hong Kong)

**Metabolic Control of Insulin Secretion**
Dr. Kenneth King-Yip CHENG
*Department of Medicine, The University of Hong Kong, Hong Kong*

**Incretin-Based Therapies for Type 2 Diabetes: Focus on East Asian Perspectives and Hypoglycemia**
Dr. Daisuke YABE
*Division of Diabetes and Endocrinology, Kansai Electric Power Medical Research Institute, Japan*

**Insulin Deficiency in the Asian Phenotype for Diabetes**
Prof. Kun-Ho YOON
*Department of Endocrinology and Metabolism, Seoul St. Mary's Hospital, The Catholic University of Korea, Korea*

**Symposium 2** — *N101A*
**Vascular Biology and DM Complications**
*Chairpersons:*
Prof. Doo-Man KIM (Korea)
Prof. Yukio TANIZAWA (Japan)

**Novel Targets in Endothelial Dysfunction**
Prof. Yu HUANG
*School of Biomedical Sciences, The Chinese University of Hong Kong, Hong Kong*

**Novel Targets for Lipid Disorders - PCSK9 and Beyond**
Prof. Brian TOMLINSON
*Department of Medicine and Therapeutics, The Chinese University of Hong Kong, Hong Kong*

**Genetics of Diabetic Complications**
Prof. Ronald MA
*Department of Medicine and Therapeutics, The Chinese University of Hong Kong, Hong Kong*

**13:45 – 15:45**

**AHKDN: Session on DM Education** — *N104-105*
**Advancing Practice in Diabetes: Nurse-Patient Partnership**
*Chairpersons:* Ms. Tracy Sau-Lin LAU, Ms. Eva Ching-Yee KAN (Hong Kong)

Ms. Veronica HUNG, *New Territories West Cluster, Hospital Authority, Hong Kong*
Ms. Yin WONG, *Family Medicine and General Out- Patient Clinic, Queen Elizabeth Hospital, Hong Kong*
Ms. Brenda LIM, *Immediate Past President and Current Vice President (Special Project), Association of Diabetes Educators (Singapore), Singapore*
Ms. Qingqing LOU, *Health Education, Jiangsu Province Hospital, China*
Prof. Xiaohui GUO, *Department of Endocrinology, First Hospital, Peking University Health Science Center, Peking University, China*
Ms. Hui-Fang WANG, *Diabetes Institute, Changhua Christian Hospital, Taiwan*
Ms. Piu-San KONG, *Department of Medicine, Centro Hospitalar Conde de São Januário, Macau*

*This session is co-organized with the Association of Hong Kong Diabetes Nurses (AHKDN)*

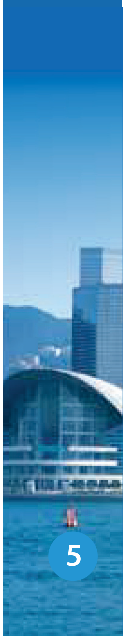

| | | |
|---|---|---|
| 15:15 – 15:45 | Health Care Industry Exhibition and Coffee Break (Sponsored by Novo Nordisk) | *N106-108* |
| | Poster Viewing | *N109-112* |
| 15:45 – 16:30 | **Plenary Lecture 2: The Masato Kasuga Award for Outstanding Scientific Achievement Presentation and Lecture**<br>*Chairpersons:* **Prof. Takashi KADOWAKI (Japan), Prof. Susumu SEINO (Japan)**<br><br>**Clarification of Molecular Mechanisms of Type 2 Diabetes Using Genetic Engineering Techniques in Mice**<br>**Dr. Naoto KUBOTA**<br>*Department of Clinical Nutrition Therapy, The University of Tokyo, Japan* | *N101B* |
| 16:30 – 17:45 | **Symposium 3** *N101B*<br>**Obesity and Diabetes**<br>*Chairpersons:*<br>**Prof. Nobuya INAGAKI (Japan)**<br>**Prof. Alice KONG (Hong Kong)**<br><br>**Effects of Nutritional Factors on Obesity and related Cardiometabolic Diseases in Chinese**<br>**Prof. Xu LIN**<br>*Shanghai Institutes for Biological Sciences, Chinese Academy of Sciences, China*<br><br>**Gut Microbiota-Derived Metabolites Shape Host Physiological Homeostasis**<br>**Dr. Shinji FUKUDA**<br>*Institute for Advanced Biosciences, Keio University, Japan*<br><br>**Metabolic Surgery for Type 2 Diabetes – Who, When and How?**<br>**Dr. Simon Kin-Hung WONG**<br>*Department of Surgery, Prince of Wales Hospital, Hong Kong* | **Symposium 4** *N101A*<br>**Molecular Insights in Diabetes**<br>*Chairpersons:*<br>**Prof. Linong JI (China)**<br>**Prof. Kathryn TAN (Hong Kong)**<br><br>**Genetic Determinants of Type 2 Diabetes and Obesity in Chinese**<br>**Prof. Cheng HU**<br>*Shanghai Diabetes Institute, Shanghai Jiao Tong University Affiliated Sixth People's Hospital, China*<br><br>**NFIA Controls the Brown Fat Gene Program by Co-Localizing with PPARγ at Cell-Type-Specific Enhancers**<br>**Dr. Hironori WAKI**<br>*Department of Diabetes and Metabolic Diseases, The University of Tokyo, Japan*<br><br>**The FGF21-Adiponectin Axis in Prevention of Obesity-Related Cardiometabolic Syndrome**<br>**Prof. Aimin XU**<br>*Department of Medicine and Department of Pharmacology and Pharmacy, The University of Hong Kong, Hong Kong* |
| 17:45 – 19:00 | **AASD Round Table Discussion for Diabetes Guideline in Asia** | *N101B* |
| 19:00 – 21:30 | **Faculty Dinner (By Invitation)** | |

### 22 November (Sunday)

**Venue: N101-112, Level 1, Hong Kong Convention and Exhibition Centre (HKCEC)**

**08:30 – 09:00**

Registration — *N101 Foyer*

**09:00 – 10:00**

**Oral Presentations (Basic)** — *N101B*
*Chairpersons:*
**Dr. Kathy LUI (Hong Kong)**
**Prof. Ronald MA (Hong Kong)**

**Oral Presentations (Clinical)** — *N101A*
*Chairpersons:*
**Dr. Jason NG (Hong Kong)**
**Dr. Yu-Cho WOO (Hong Kong)**

**Oral Presentations (HKSEMR)** — *N104-105*
*Chairpersons:*
**Dr. Philip Chi-Ngong CHIU (Hong Kong)**
**Dr. Gida WONG (Hong Kong)**

**10:00 – 10:45**

**Plenary Lecture 3: The Pan Xiaoren Distinguished Research Award for Epidemiology of Diabetes in Asia Presentation and Lecture** — *N101B*
*Chairpersons:* **Prof. Susumu SEINO (Japan), Prof. Linong JI (China)**

**From Epidemiology to Personalized Medicine in Diabetes**
**Prof. Juliana Chung-Ngor CHAN**
*Department of Medicine and Therapeutics, The Chinese University of Hong Kong, Hong Kong*

**10:45 – 11:15**

Health Care Industry Exhibition and Coffee Break — *N106-108*

Poster Viewing — *N109-112*

**11:15 – 12:30**

**Symposium 5** — *N101B*
**Update on Treatment Guidelines in Diabetes**
*Chairpersons:*
**Prof. Nigishi HOTTA (Japan)**
**Dr. Peter TONG (Hong Kong)**

**Glycaemic Targets**
**Dr. Shih-Yi LIN**
*Division of Endocrinology and Metabolism, Taichung Veterans General Hospital, Taiwan*

**BP Targets**
**Prof. Bernard CHEUNG**
*Department of Medicine, The University of Hong Kong, Hong Kong*

**Lipid Targets**
**Prof. Kathryn TAN**
*Department of Medicine, The University of Hong Kong, Hong Kong*

**Symposium 6** — *N101A*
**Recent Advances in Endocrine Research**
*Chairpersons:*
**Prof. Alice WONG (Hong Kong)**
**Prof. Anderson O. L. WONG (Hong Kong)**

**Novel Pituitary Actions of TAC3/ NK3R System**
**Prof. Anderson O. L. WONG**
*School of Biological Sciences, The University of Hong Kong, Hong Kong*

**Clinical Utility of Anti-Mullerian Hormone Measurement**
**Dr. Raymond Hang-Wun LI**
*Department of Obstetrics and Gynaecology, Queen Mary Hospital, Hong Kong*

**Stimulation of Bone Formation by Local Delivery of Minerals**
**Dr. Kelvin YEUNG**
*Department of Orthopaedics and Traumatology, The University of Hong Kong, Hong Kong*

**12:30 – 13:10**

**Lunch** — *N101B*

**Presidential Poster Tour** — *N109-110*
*Chairperson:*
**Dr. Daisuke YABE (Japan)**

**Annual General Meeting of HKSEMR** — *N104-105*

**13:10 – 14:00**

**Lunch Symposium (Sponsored by AstraZeneca)** — *N101B*
**DM Expert Forum – Sharing from East to West**
*Chairpersons:* **Prof. Juliana Chung-Ngor CHAN (Hong Kong)**

**Advance DM Management – from Guideline to Clinical Practice**
**Prof. Stephan JACOB**
*University of Tübingen, Germany*

**GLP1 Case Sharing from Mainland**
**Prof. Hui FAN**
*Beijing Chaoyang Hospital, Capital Medical University, China*

**SGLT2 Case Sharing from Hong Kong**
**Dr. Norman CHAN**
*Department of Medicine and Therapeutics, The Chinese University of Hong Kong, Hong Kong*

**Latest Clinical Experience from Germany**
**Prof. Stephan JACOB**
*University of Tübingen, Germany*

| Time | Programme | | |
|---|---|---|---|
| 14:00 – 14:45 | **President's Lecture**<br>**Plenary Lecture 4: Insights on the Roles of Adipokines in Clinical Diabetes**<br>*Chairperson:* Prof. Yutaka SEINO (Japan)<br><br>Prof. Karen LAM<br>*Department of Medicine, The University of Hong Kong, Hong Kong* | | *N101B* |
| 14:45 – 15:00 | Health Care Industry Exhibition and Coffee Break | | *N106-108* |
| 15:00 – 16:15 | **Symposium 7** *N101B*<br>**DM Co-Morbidities and Their Management**<br>*Chairpersons:*<br>Dr. Ying-Wai NG (Hong Kong)<br>Dr. Wing-Yee SO (Hong Kong)<br><br>**Diabetes and Depression - What is the Link?**<br>Dr. Andrea LUK<br>*Department of Medicine and Therapeutics, The Chinese University of Hong Kong, Hong Kong*<br><br>**Renal Complications in DM**<br>Prof. Per-Henrik GROOP<br>*University of Helsinki, Finland*<br><br>**Ubiquitous Healthcare in Patients with Diabetes**<br>Prof. Soo LIM<br>*Department of Internal Medicine, Seoul National University Bundang Hospital, Korea* | **Symposium 8**<br>**Controversies in the Management of Endocrine Disorders**<br>*Chairpersons:*<br>Dr. Tai-Pang IP (Hong Kong)<br>Dr. Sau-Cheung TIU (Hong Kong)<br><br>**Vitamin D in Endocrine and Cardiometabolic Diseases**<br>Dr. Ching-Lung CHEUNG<br>*Department of Pharmacology and Pharmacy, The University of Hong Kong, Hong Kong*<br><br>**Testosterone Replacement: Facts or Fiction**<br>Dr. Ka-Kui LEE<br>*Department of Medicine, The University of Hong Kong, Hong Kong*<br><br>**Management of Abnormal Thyroid Function in Pregnancy**<br>Prof. Wing-Hung TAM<br>*Department of Obstetrics and Gynaecology, The Chinese University of Hong Kong, Hong Kong* | *N101A* |
| 16:15 – 16:30 | **Presentation of Young Investigator Awards**<br>**Closing Ceremony** | | *N101B* |
